# Three-year clinical performance of two indirect composite inlays compared to direct composite restorations

**DOI:** 10.4317/medoral.18491

**Published:** 2013-03-25

**Authors:** Nurcan Ozakar-Ilda, Yahya O. Zorba, Mehmet Yildiz, Vildan Erdem, Nilgun Seven, Sezer Demirbuga

**Affiliations:** 1 Assistant Professor, Department of Restorative Dentistry, Faculty of Dentistry, Ataturk University, Erzurum, Turkey; 2 Associate Professor, Department of Restorative Dentistry, Faculty of Dentistry, Erciyes University, Kayseri, Turkey; 3 Professor, Department of Restorative Dentistry, Faculty of Dentistry, Ataturk University, Erzurum, Turkey; 4 Research Assistant, Department of Restorative Dentistry, Faculty of Dentistry, Ataturk University, Erzurum, Turkey; 5 Research Assistant, Department of Restorative Dentistry, Faculty of Dentistry, Erciyes University, Kayseri, Turkey

## Abstract

Objective: Despite the incremental build-up of resin composite restorations, their polymerization shrinkage during curing presents a serious problem. Indirect composite resin systems represent an alternative in overcoming some of the deficiencies of direct composite restorations. The hypothesis of the present study states that the clinical performance of restorations may be affected by different generation and application techniques. Study Design: Sixty restorations (20 DI system (Coltène/Whaledent AG, Altstätten, Switzerland) composite inlays, 20 Tescera ATL system (BISCO Inc. Schaumburg, Illinois, USA) composite inlays, and 20 direct composites) were applied to premolar teeth in 49 patients. Restorations were clinically evaluated by two examiners. Data were analyzed using the Kruskal-Wallis, Mann-Whitney U, Wilcoxon Signed Ranks, and X2 tests. Results: The Tescera ATL system performed significantly better than both direct composite restorations (p<0.001) and DI system (p<0.05). Conclusion: Within the limitations of this 3-year clinical study, indirect resin restorations showed better scores than direct restorations. In addition, the Tescera ATL system was found to be more successful than the DI system and direct composite restorations.

** Key words:**Composite, inlay, direct composite restorations, indirect composite restorations.

## Introduction

Advances in restorative dentistry and increases in patient expectations regarding aesthetics have led to demands for non-metallic, tooth-colored restorations in the posterior region (1). Indirect composite resin systems represent an alternative to overcome some deficiencies of direct composite restorations.

The composition of indirect composite resin systems is similar to that of direct systems, differing by the use of various methods of additional polymerization, which cause a higher radical conversion. These additional polymerization procedures can involve photo-activation, heat, pressure, and a nitrogen atmosphere (2). Post curing at high temperature results in a higher stress relaxation and degree of conversion compared to the directly placed light-cured composite restoration. Moreover, polymerization shrinkage takes place outside the mouth, thus limiting the shrinkage to that of the thin luting cement layer (3). In comparison to direct composite restorations, indirect adhesive restorations are believed to exhibit better proximal contact, occlusal morphology and marginal compatibility (4).

The first generation of laboratory composites was developed in the 1980s as an alternative for clinicians to overcome some inherent deficiencies of direct composites restorations, including polymerization shrinkage, inadequate polymerization in deep inter-proximal areas and restoration of proximal contacts and contour (5). In spite of their secondary curing (by heat, light, pressure, or argon laser), the first generation laboratory inlay composite resins exhibit low levels of flexural strength (60-80 MPa) and elastic modulus (2.0-3.5 GPa), a resin volume percentage higher than 50% and high abrasive wear levels in conjunction with low levels of inorganic filler contents (6). Because of these disadvantages, in the early 1990s a second generation of laboratory-processed resin composites, or polyglass materials, was developed. The manufacturer advocated these materials could be used for a wide range of fixed prosthodontic applications such as inlays, onlays, veneering, metal-free single unit crowns, and short span anterior bridges (5). These materials can be classified as microhybrid composites with higher inorganic fillers of approximately 66% by volume. This situation results in improved mechanical properties with flexural strength between 120-160 MPa and elastic modulus of 8.5-12 GPa (7).

DI system (Coltène/Whaledent AG, Altstätten, Switzerland) is a first-generation laboratory composite system that consists of a hybrid composite containing fine-particle glass filler and a DI 500 heat/light cure oven required for advanced polymerization. Tescera ATL system (BISCO Inc., Schaumburg, Illinois, USA) is a second-generation laboratory composite system that, in addition to heat and light, utilizes air pressure for polymerization. The system consists of a microhybrid composite and an Indirect Aqua Thermal Light Polymerization unit for processing indirect composites in an oxygen-free environment.

Although laboratory-processed indirect restorations have been previously reported to offer minimal polymerization shrinkage and maximum aesthetic satisfaction together with good mechanical and biological functioning (5), in general, there is insufficient data in the literature regarding characteristics and clinical performance of laboratory-processed resin materials.

This study aimed to assess the 3-year clinical performances of DI system and Tescera ATL indirect composite inlays when used for restoration of premolar teeth. The null hypothesis in this study was that the clinical performance of composite inlays may be affected by different generation and application techniques.

## Material and Methods

 Table 1 lists the brand names, manufacturers, polymerization type, and content of the restorative materials used in the study. Twenty direct composite restorations, 20 DI system indirect composite inlay restorations and 20 Tescera ATL system indirect composite inlay restorations were applied to the upper and lower premolars of 49 non-smoking patients (28 male, 21 female; mean age: 32) by the same researcher. Approval was obtained from the university ethics committee, and treatment plans were approved by the patient. For indirect restorations, only vital premolar teeth in occlusion, with at least one proximal contact with an adjacent tooth and requiring large cavities for which the isthmus width extended two thirds of the intercuspal distance were included in the study. Teeth with preoperative symptoms or requiring restorations with subgingival edges 3 mm or more below the cemento-enamel junction were excluded from the study. Small- and medium-sized defects, namely isthmus width smaller than one-half to two-thirds of the intercuspal distance, were treated with an incrementally placed direct composite restoration.

**Table 1:**
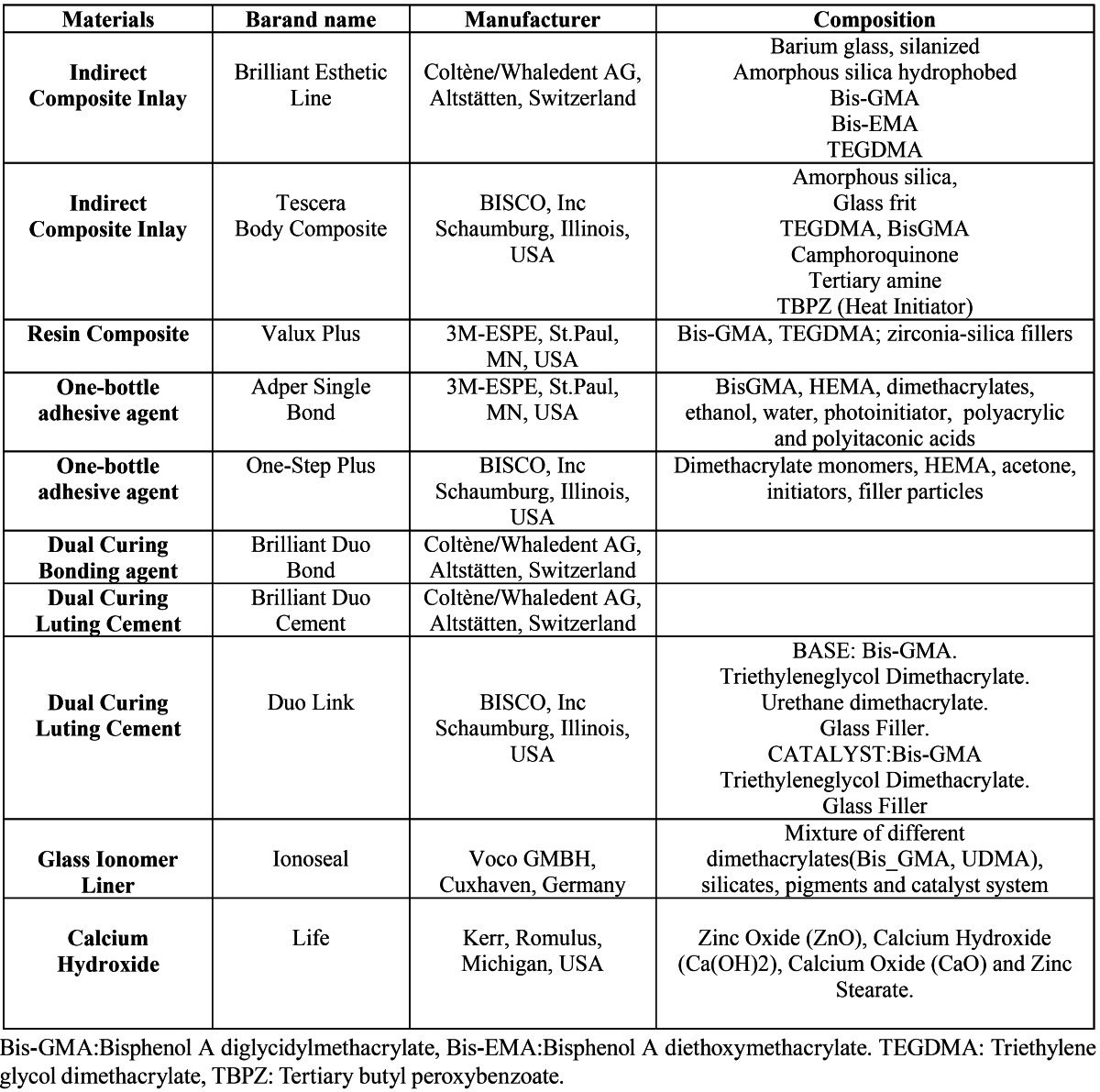
Manufacturers and specifications of materials used in the present study.

All inlay cavities were prepared according to the common principles for adhesive inlays. Class II cavity preparation was initiated with 80 mm diamond grit tapered fissure burs (Diatech, Coltène/Whaledent AG, Altstätten, Switzerland) to obtain a convergence angle of 10°-12° between opposing walls. Internal line and point angles were rounded, and butt joints were used for enamel margins. The pulpal floor was shaped to obtain an occlusal thickness between 1.5-2 mm, and all undercuts were removed. Cavities were finished with 25 mm diamond grit tapered fissure burs (Diatech, Coltène/Whaledent AG, Altstätten, Switzerland).

Restorative treatment was performed under local anesthesia. Patients were fitted with a rubber dam. In cases where the cavity was deep, a calcium hydroxide lining (Life, Kerr, Portland, Oregon, USA) and a thin layer of glass-ionomer liner (Ionobond, Voco, Cuxhaven, Germany) were applied, respectively. Complete arch impressions were taken using a polyether impression material (Impregum F, 3M-ESPE, St Paul, MN, USA), and temporary restorations were placed using an eugenol-free temporary cement (Provicol, Voco, Cuxhaven, Germany). All inlays were fabricated in the same commercial laboratory by the same technician.

All inlays were definitively inserted within two weeks after fabrication. The temporary restorations were removed, and prepared teeth were cleaned using a rubber cup and pumice slurry. Enamel cavity margins were acid-etched with 35% orthophosphoric acid (3M ESPE Dental Products, St. Paul, MN, USA) scrubbed for 15-20 seconds, and rinsed by thoroughly spraying with water for 20-25 seconds. The excess water was blotted using a cotton pellet.

For applying the DI inlay system, the inner surface of the restoration was sandblasted with 50 µm alumina particles and rinsed with water. A dual-cured bonding agent (Brilliant Duo Bond, Coltène/Whaledent AG, Altstätten, Switzerland) was applied for 15 seconds with gentle agitation, and disbursed using a gentle stream of compressed air without light-curing. A clear plastic matrix band and light-reflecting wedges (Luci Wedges, Hawe Neos, manufactured for Coltene) were inserted, and a dual-curing luting composite (Brilliant Duo Cement, Coltène/Whaledent AG, Altstätten, Switzerland) was applied to the cavity and/or the inside of the inlay using a disposable brush. Inlays were quickly inserted using moderate pressure, and any major excess cement was removed using an explorer and dental floss. Inlay surfaces were light-activated for 40 seconds each using a light emitting diode curing unit (LED, Elipar Free Light 2, 3M ESPE Dental Products, St. Paul, MN, USA). Following curing, occlusion and articulation were carefully checked, and interproximal contacts were controlled with dental floss. Inlays were finished under water-cooling using fine-particle finishing diamonds, carbide finishing burs, polishing strips (Soflex, 3M-ESPE, Dental Products, St. Paul, MN, USA) and a composite finishing polishing kit (Enhance, Dentsply-Caulk, Milford, Del, USA).

For applying Tescera ATL inlay system, two drops of One-step plus (BISCO Inc., Schaumburg, Illinois, USA) was dispensed into a mixing well. Two generous coats of adhesive were applied for 15 seconds with gentle agitation and gently air thinned for 5 seconds to evaporate solvent and then light cured for 10 seconds with LED. The inner surface of the restoration was sandblasted with 50 µm alumina particles and rinsed with water. Two coats of composite activator (BISCO, Inc., Schaumburg, Illinois, USA) were applied to the inner surface of restoration and then air-dried. A dual-cure resin cement was applied (Duo Link, BISCO, Inc., Schaumburg, IL, USA) to the cavity and/or the inside of the inlay using a disposable brush. The inlays were cemented, controlled, and finished as described above.

Direct composite restorations were produced in a class II cavity. The preparation was performed in the manner described above for adhesive inlays; however, preparation was limited to the removal of decay so as to preserve sound tooth structure. Two consecutive coats of adhesive (Single Bond, 3M-ESPE, St. Paul, MN. USA) were applied for 15 seconds with gentle agitation and gently air thinned for 5 seconds to evaporate solvent then light cured for 10 seconds with LED as to manufacturer’s instructions. Composite resin (Valux Plus, 3M-ESPE, St. Paul, MN, USA) was placed incrementally, with each layer light-cured for 40 seconds using LED. Finishing and polishing were performed as described above.

At the initial recall examination (after one week) and after a 3-year period, Modified U.S. Public Health Service (USPHS) criteria was used by two independent, experienced examiners using mirrors and probes to analyze the degree of quality, according to the description in a study by Kramer N-Frankenberger R (8) and Ernst CP et al. (9) ( Table 2). Alpha and Bravo scores indicate “excellent” and “clinically acceptable” results, while Charlie and Delta scores indicate “clinically not acceptable” (9). The differences were discussed in cases where there was an initial disagreement between the examiners and an ultimate decision was reached by consensus.

**Table 2:**
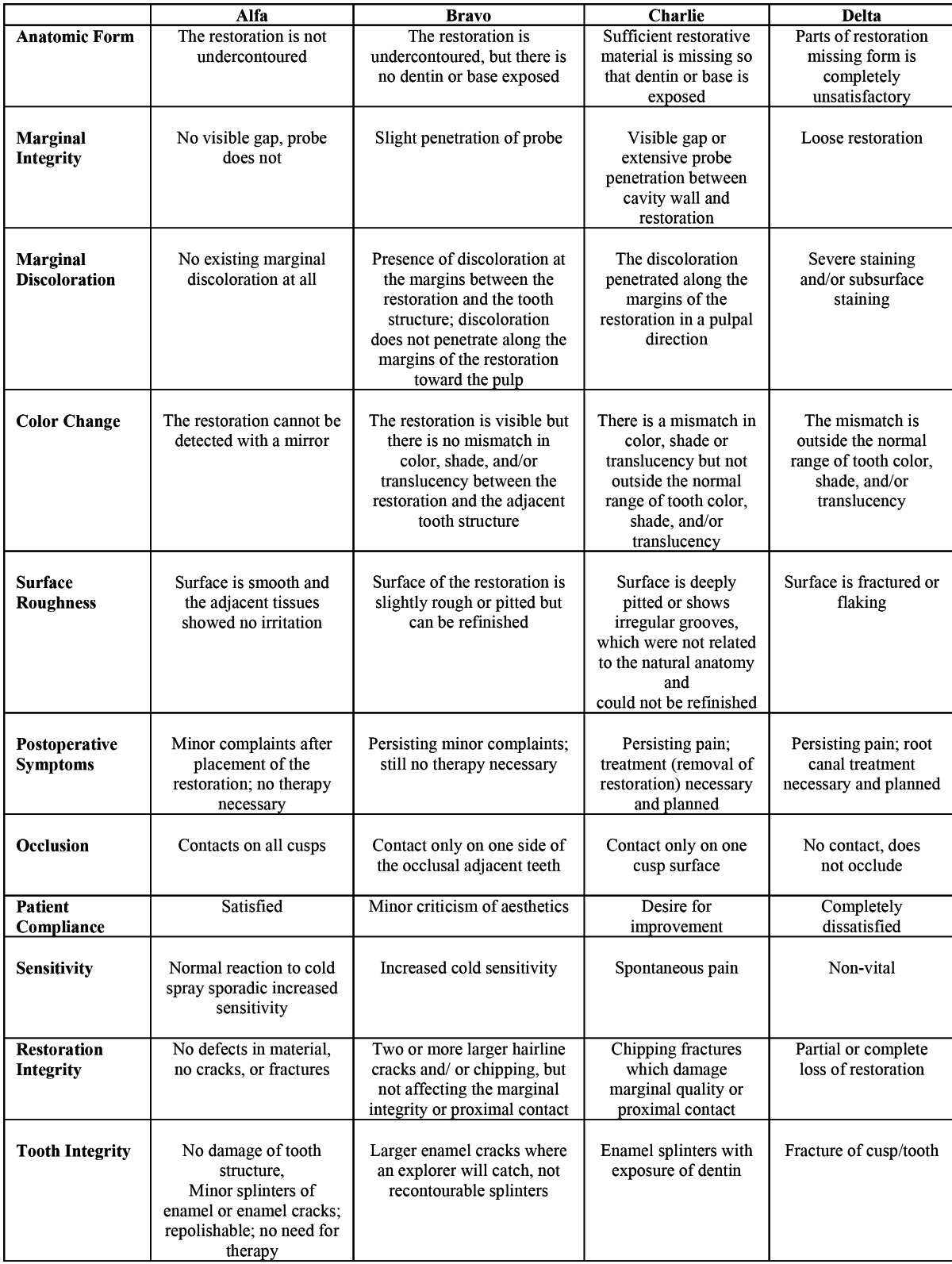
Descriptive criteria used for scoring restoration quality.

-Statistical analysis

Statistical analysis was performed using the SPSS software (Version 16.0, SPSS, Chicago, IL, USA). Differences between groups were evaluated using Kruskal-Wallis and Mann-Whitney U tests, and changes between the initial and 3-year assessments were evaluated using the Wilcoxon Signed Ranks test. A level of p<0.05 was considered significant.

## Results

After 36 months, all restorations were available for clinical evaluation (Recall rate: 100%). Clinical evaluations for each group at initial recall and after 3 years are presented in  Table 3.

**Table 3:**
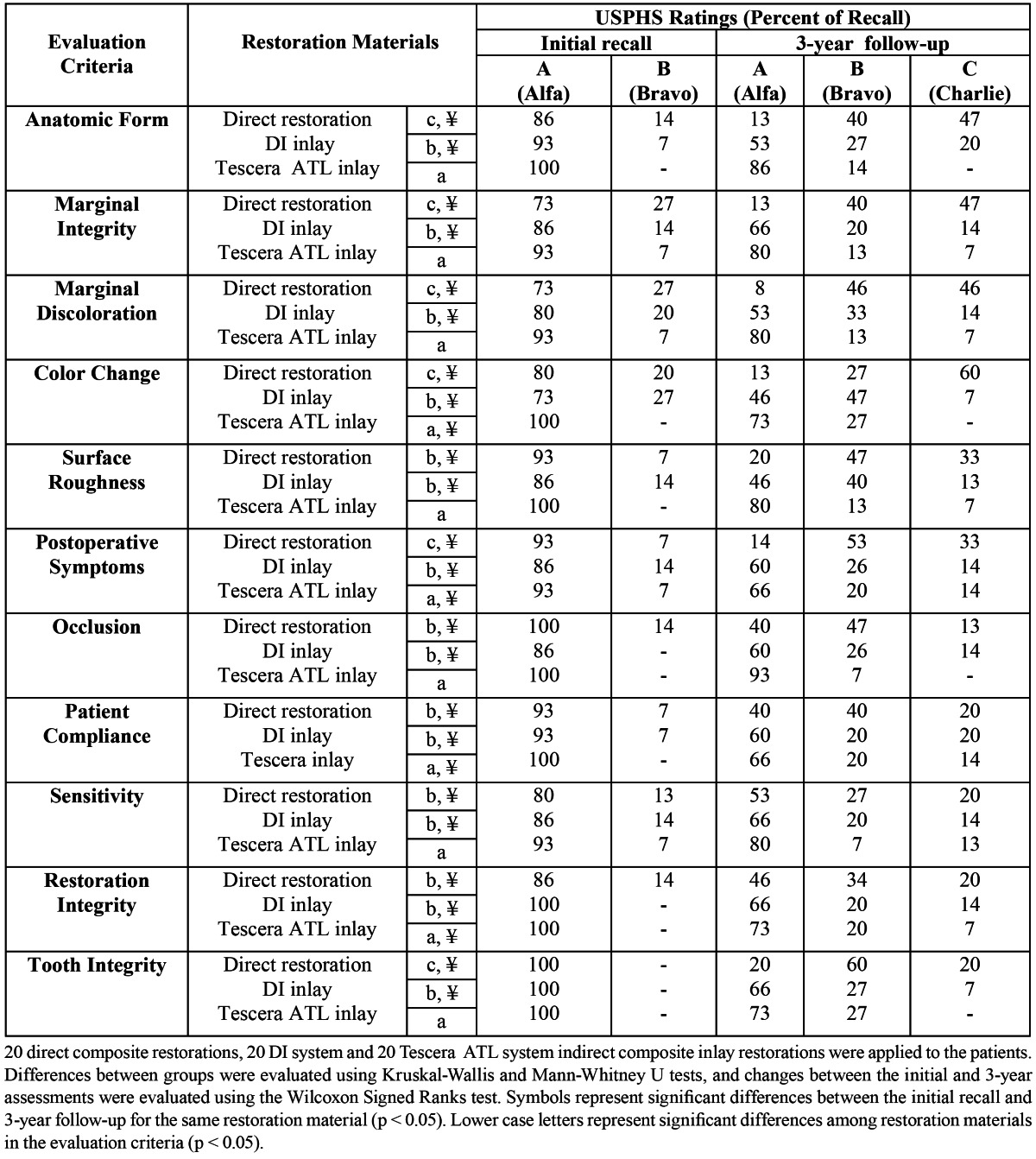
Modified USPHS ratings of restorations at initial recall and after 3 years.

After 3 years, in terms of all 11 criteria evaluated, the Tescera ATL inlay system was found to perform significantly better than both direct composite restorations (p<0.001) and the DI inlay system (p<0.001 for color match, p<0.05 for all other criteria). The DI inlay system was also found to perform significantly better (p<0.05) than direct composite restorations in terms of anatomic form, marginal integrity, marginal discoloration, color change, post-operative symptoms, and tooth integrity. However, no significant differences were found between the DI system inlays and direct restorations in terms of surface roughness, occlusion, patient complaints, sensitivity, and restoration integrity.

Significant differences were found between the initial evaluations and the 3-year evaluations for all criteria (p<0.05 for sensitivity and occlusion, p<0.001 for all other criteria) for all 3 groups tested ( Table 3). However, at the end of the 3-year follow-up, no Delta (insufficient/poor) scores were observed for any of the evaluation criteria in any of the groups.

The Wilcoxon test proved a statistically significant differences in the evaluation of anatomic form, marginal integrity, marginal discoloration, color change, surface roughness, postoperative symptoms, tooth integrity (p=0.001), occlusion, patient compliance (p=0.005), restoration integrity (p=0.004), and sensitivity (p=0.014) between the initial and 3-year evaluations for direct composite restorations. All criteria from the DI group were statistically and significantly different (p <0.05) in terms of marginal integrity, surface roughness, color change, and sensitivity (p=0.025), anatomic form, postoperative symptoms, marginal discoloration, and occlusion (p=0.014), patient compliance (p=0.023), and restoration integrity, and tooth integrity (p=0.034) between the initial and 3-year evaluations. Tescera ATL group did not present significant differences between the initial and 3-year evaluations except for color change, post-operative symptoms (p=0.046), patient complaints, and restoration integrity (p=0.025).

The main reasons for failure in the direct restoration group were anatomic form, marginal integrity, marginal discoloration, and color change. The Coltene DI system group was unsuccessful in terms of anatomic form and postoperative symptoms.

In Tescera ATL group, the postoperative symptoms and patient compliance criteria had higher C scores compared to the other criteria.

## Discussion

The null hypothesis “the clinical performance of composite inlays may be affected by different generation and application” was confirmed.

Due to the increasing use of composites and the number of new resin brands, it is important for dentists to be aware of the probable longevity and likely modes of failure in composite restorations. This information is best obtained from randomized controlled trials conducted clinically and in the laboratory (10). Although the majority of inlays are applied to molar teeth, the use of indirect restorations may be more appropriate and more beneficial in premolar teeth, which are less prone to occlusal abrasion than posterior teeth and are also more important in terms of facial aesthetics (11). Moreover, easy access to this region permits the clinician to maintain better control of the technique. For this reason, this study focused only on premolar teeth.

A 3-year clinical follow-up with modified USPHS criteria produced a success rate of 93% for Tescera ATL system indirect inlays, 86% for DI system indirect inlays, and 67% for direct composite fillings. These results are in line with previous reports of success rates of 80 -100% for composite inlays following similar observation periods (12-14) ( Table 4). Previous studies showed that the physical and mechanical properties of resin composites are greatly affected by the degree of conversion (DC) in the cross-linked polymeric system (15). This may affect the clinical performance of restorative material. Both DI and Tescera ATL indirect inlay systems use heat and light as part of the post-cure process; however, in the Tescera ATL indirect inlay system, post-cure heat and light are applied under pressure, which results in better mechanical properties and adhesion between layers. The reason for the Tescera ATL system’s good clinical performance may be use of aqua thermal light polymerization unit in an oxygen-free environment. Because the post-curing procedure took place in a pressurized environment, the superior aesthetic and mechanical properties exhibited by the Tescera inlays likely attributed to higher wear-resistance. In addition, Tescera’s good wear-resistance performance may also be related to the micro-particles in the filler.

**Table 4:**
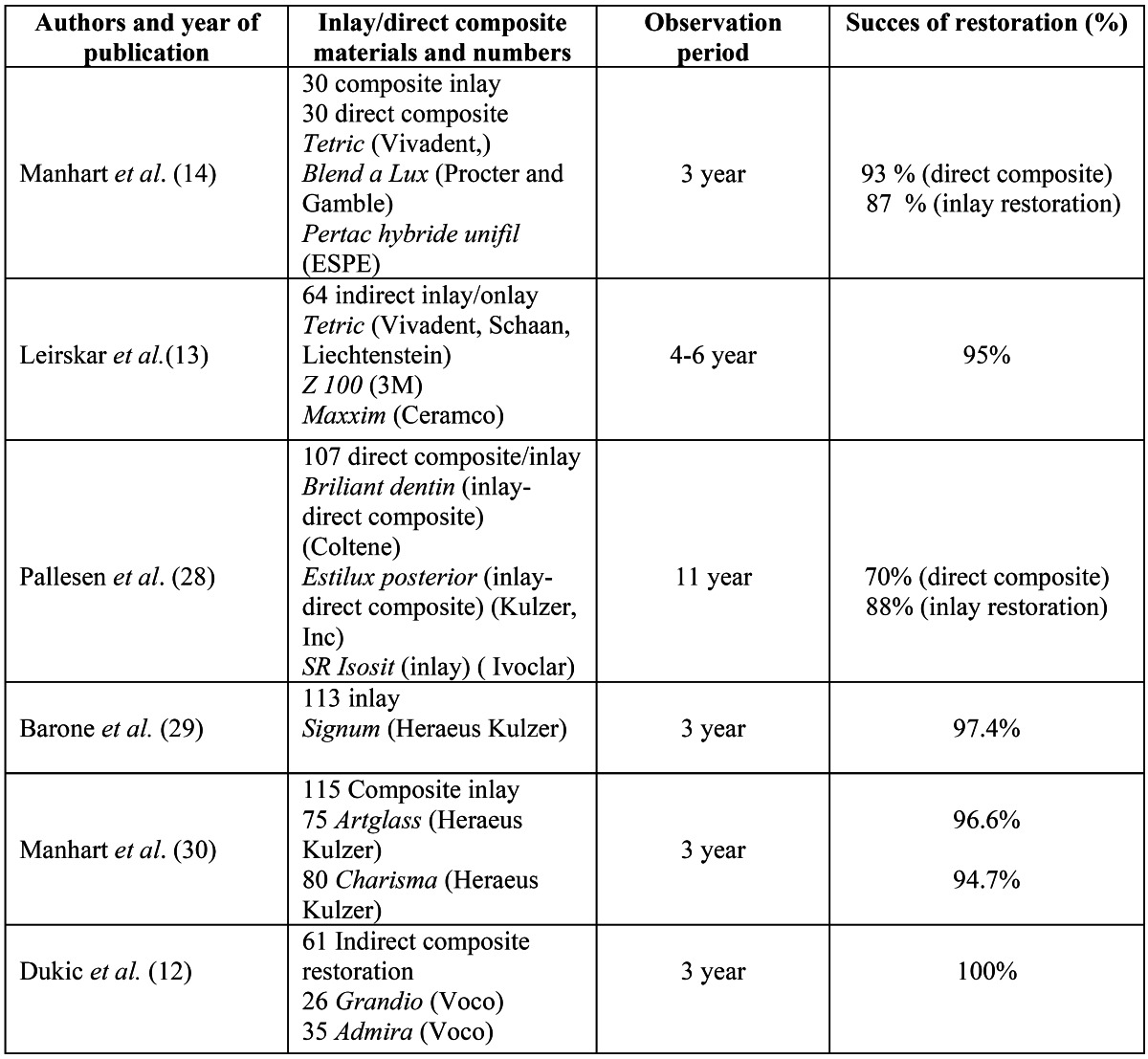
Success rates of inlays and direct composite restorations.

In the present study, both indirect restoration systems performed better than direct composite restorations in terms of anatomic form, marginal adaptation, marginal discoloration, color change, and postoperative symptoms. These findings contrast with those of a different five-year clinical study (16), which showed no differences in survival rates among direct and indirect resin composite and ceramic inlay restorations. Wassel compared the success rate between direct inlays and direct conventional composite restorations, showed that the direct inlay technique has no clinical advantage when compared with the direct conventional technique (17). The difference in findings may be due to the differences in follow-up periods between the studies and to the selection of different materials.

Terry and Touati suggested that after photoactivation, additional treatments such as heat and/or light cure and mechanical polishing lead to an increase in degree of conversion, improved mechanical properties, color stability, and reduction of wear (18).

Klymus et al. reported that composites polymerized under high temperatures (belleGlass and Targis) have higher flexural strength and modulus of elasticity than composites polymerized by light (Artglass and Solidex) (19). The findings of present study confirmed these conclusions.

In contrast to the findings of our study, a 3-year clinical study by Wendt et al. comparing indirect, light-polymerized composite resin inlays with indirect, heat-polymerized composite resin inlays found no differences in terms of wear resistance (20). However, the same study showed heat-polymerized inlays to be more successful in terms of marginal integrity and marginal discoloration (interfacial staining), which is in line with the findings of our study.

In a previous study, the authors observed that composite inlays reduced postoperative sensitivity and microleakage and provided better sealing and clinical performance when compared to direct restorations after 3 years of follow-up. (14). Liberman et al. concluded that the inlay technique provides better marginal coverage than direct placement (21). However, Dukuia et al. concluded that indirect systems only provide better sealing than direct composites in enamel surface. (22). On the contrary, Soares et al. found that there were no significant differences between direct and indirect techniques for the cervical finishing line in enamel, but for the finishing line in dentin, the indirect technique allowed less microleakage than the direct technique (23). In the present study, indirect restorations showed better marginal integrity when compared the direct technique. In addition, after a 3-year clinical follow-up, the Tescera ATL system showed superior marginal integrity when compared the others.

A number of factors may play a role in the post-operative discoloration of restorations, including chemical reactions within the resin matrix or the material’s surface structure and the interaction between the organic matrix and filler particles (24). Fillers also enhance aesthetics and improve handling properties, and the modification of filler size and morphology results in improved mechanical properties and aesthetics compared with earlier composite materials (25). The result of this study showed that post curing increased color stability. Also, the Tescera ATL system was better than the other with regard to color change. Because the Tescera composite includes small inorganic microparticles, it may provide better surface polishing and help to remove foods and accumulated plaque, which would help to maintain long term-color stability.

Previous studies showed that both direct and indirect restorations provide satisfactory clinical performance; also, there was no significant difference between them in the short term (26,27). A 3-year period may not be considered sufficient for drawing definitive conclusions on the performance of direct and indirect restoration techniques; however, within the limitations of the results of the present study, an indirect composite may offer a good alternative to direct restorations for long-term clinical performance. In time, the results of 5-year follow-up examinations are expected to yield more clinically-relevant information.
